# NOX2/NLRP3-Inflammasome-Dependent Microglia Activation Promotes As(III)-Induced Learning and Memory Impairments in Developmental Rats

**DOI:** 10.3390/toxics13070538

**Published:** 2025-06-26

**Authors:** Linlin Zhang, Yuyao Xiao, Dan Wang, Xuerong Han, Ruoqi Zhou, Huiying Zhang, Kexin Zhu, Junyao Wu, Xiance Sun, Shuangyue Li

**Affiliations:** 1 School of Public Health, Dalian Medical University, No. 9 West Section Lvshun South Road, Dalian 116044, China; 15998640631@163.com (L.Z.); lndllyh@126.com (D.W.); 19204181796@163.com (R.Z.); zhang241640@163.com (H.Z.); a949363877@163.com (K.Z.); wjy237630@126.com (J.W.); 2 College of Traditional Chinese Medicine, Beijing University of Chinese Medicine, No. 6 Wangjing South Road, Beijing 102401, China; 20230121122@bucm.edu.cn; 3 Dalian Municipal Center for Disease Control and Prevention Environmental Health Department, No. 151, Yueling West Street, Dalian 116013, China; hanxuerong85@126.com

**Keywords:** inorganic arsenic, microglia, NLRP3 inflammasome, NOX2, learning and memory

## Abstract

Inorganic arsenic [As(III) and As(V)] is a pervasive environmental contaminant in groundwater systems, early-life exposure to which is associated with an impaired cognitive ability and an increased risk of neurobehavioral disorders. Although the effect of As(III) on the neurons is well studied, the involvement of the microglia remains unclear. In this study, the effects of sodium arsenite (NaAsO_2_) on microglial activation and the underlying NLRP3 inflammasome mechanism were determined. Pregnant rats were gavaged with NaAsO_2_ (0, 1, 4, and 10 mg/kg body weight), which dissociates in aqueous solutions into bioactive arsenite species [As(OH)_3_], from gestational day 1 (GD1) to postnatal day 21 (PND21). The results showed that As(III) induces learning and memory impairments and microglial activation in the hippocampus of offspring rats (PND21). Increased expression of NLRP3, the activation of caspase-1, and the production of interleukin-1β were observed in both the hippocampus of As(III)-exposed offspring rats and As(III)-exposed microglial BV2 cells under culture conditions. Interestingly, blocking the NLRP3 inflammasome using MCC950 mitigated its activation. Furthermore, inhibition of NADPH oxidase 2 (NOX2) using apocynin or specific siRNA significantly reduced As(III)-induced microglial NLRP3 inflammasome activation. In addition, inactivation of the microglial NLRP3 inflammasome or NOX2 markedly rescued As(III)-induced neurotoxicity in the hippocampal HT22 cells. Taken together, this study reveals that NOX2/NLRP3-inflammasome-dependent microglial activation promotes As(III)-induced learning and memory impairments in developmental rats.

## 1. Introduction

Arsenic is a metalloid that is ubiquitously distributed in nature. Globally, approximately 200 million individuals are at risk of inorganic arsenic [iAs = As (III) and As(V)] exposure, exceeding the safety threshold (10 µg/L) established by the World Health Organization (WHO) [1]. Environmental iAs contamination persists as a major global public health challenge, particularly affecting Asian countries, which bear 94% of the worldwide burden [1]. Inorganic As exposure is associated with diverse pathologies, including cardiovascular disorders, dermatological lesions, and malignancies [2]. Notably, neurotoxicity emerges as a critical concern, contributing to memory impairments and cognitive deficits in exposed populations [3].

Emerging evidence underscores the neurodevelopmental toxicity of As(III), especially during critical windows of early life. As(III) can penetrate the developing nervous system through transplacental transfer, breast milk, and the immature blood–brain barrier [4,5,6]. Given the weakened resistance in developing organisms, As(III) tends to persistently accumulate in the brain [7]. Consequently, even short-term As(III) exposure during this period may inflict irreversible neural damage, thereby significantly elevating the lifelong risks of neurobehavioral disorders [8,9]. Epidemiological studies have demonstrated that early-life As(III) exposure increased the risk of intellectual disabilities, reduced intellectual quotients, and impaired neurobehavioral scores in children [7,10]. Maternal As(III) exposure has been demonstrated to induce a range of physiological and pathological alterations in offspring, thereby disrupting the structural and functional integrity of the central nervous system. However, at present, the potential mechanism behind As(III)-induced neurodevelopmental damage is still not completely understood.

Neuroinflammation is a well-established contributor to memory impairments and cognitive deficits. As the primary immune cells of the CNS, the microglia regulate homeostasis but also drive neurotoxic inflammation when aberrantly activated. Emerging experimental evidence has shown that hippocampal microglial activation contributes to deficits in learning–memory [11]. Recent studies demonstrate that developmental As(III) exposure induces microglial activation in the cerebral cortex and the hippocampus, accompanied by neuronal loss and synaptic structural damage [12]. These As(III)-exposed models exhibit elevated pro-inflammatory cytokines (e.g., IL-1β, TNF-α), which subsequently damage the integrity of neural circuits [13,14]. Therefore, it is essential to elucidate the mechanisms through which As(III) induces microglial activation, particularly during vulnerable developmental periods.

Emerging research highlights the pivotal role of NOD-like receptor protein 3 (NLRP3) inflammasome complexes in regulating the microglial activation associated with learning and memory deficits. The NLRP3 inflammasome, a multiprotein complex central to innate immunity, has emerged as a critical mediator of neuroinflammation and cognitive dysfunction [15]. Upon activation, NLRP3 recruits apoptosis-associated speck-like protein (ASC) and caspase-1 to facilitate the maturation and release of pro-inflammatory cytokines such as interleukin-1β (IL-1β) and IL-18 [16]. Activation of the NLRP3 inflammasome exerts pleiotropic effects on synaptic plasticity, neuronal function, and neurodevelopment [17,18,19,20]. Several toxic metals, including manganese [as MnCl_2_, Mn (II)], copper [as CuCl_2_, Cu(II)], and iron [as FeCl_3_·6H_2_O, Fe(III)], can induce NLRP3 inflammasome activation, which is associated with learning and memory deficits [21,22,23,24]. In a learning and memory impairment mouse model induced through Pb(CH_3_COO)_2_ [Pb(II)] exposure, genetic deletion of NLRP3 markedly attenuated microglial activation, which consequently led to reduced neuropathological alterations and enhanced cognitive performance [25]. In As(III)-exposed mouse models, activation of the NLRP3 inflammasome has been observed in the hippocampus [26,27]. Additionally, specific inactivation of microglial NLRP3 inflammasomes has been shown to mitigate sevoflurane-induced learning and memory impairments in rat offspring [28]. Despite these findings, the involvement of the NLRP3 inflammasome in As(III)-induced microglial activation remains to be demonstrated.

This study investigates the microglial activation mechanisms underlying gestational/lactational As(III) exposure-induced learning and memory impairments in developmental rats. Our findings demonstrate that the NLRP3 inflammasome contributes to As(III)-induced microglial activation. Pharmacological inhibition of NADPH oxidase 2 (NOX2) or genetic knockdown of NOX2 significantly ameliorated As(III)-induced NLRP3 inflammasome activation. Importantly, inhibition of the microglial NLRP3 inflammasome or NOX2 mitigated the arsenite-caused hippocampal cell impairment. These findings identify the NOX2/NLRP3 inflammasome as a critical regulator of microglia-driven learning and memory deficits, providing mechanistic insights into As(III)-related neurodevelopmental toxicity and potential therapeutic targets.

## 2. Materials and Methods

### 2.1. The Animal Model and Treatment

Specific pathogen free (SPF)-grade eight-week-old Sprague-Dawley rats, weighing 180–220 g, were obtained from the Animal Center of Dalian Medical University. The animals were housed under controlled environmental conditions (temperature: 22 ± 2 °C; 12-h light/dark cycle) with ad libitum access to standard rodent chow (SPF-grade, 18% protein, 5% fat; Changsheng Biotechnology, Shenyang, Liaoning, China) and distilled water. Female rats were paired with male rats at a 2:1 ratio for mating. Pregnancy was confirmed through the presence of vaginal plugs, designated as gestational day 1 (GD1). Pregnant rats were randomly divided into four experimental groups (n = 6 per group). Sodium arsenite (NaAsO_2_, Sigma-Aldrich, St. Louis, MO, USA, ≥90% purity) was dissolved in sterile deionized water (pH 7.0) for animal exposure. NaAsO_2_ dissociates in aqueous solutions to form arsenous acid [As(OH)_3_], which is the bioavailable trivalent arsenic species [As(III)] responsible for mammalian toxicity. All subsequent references to “arsenite exposure” denote this trivalent species. NaAsO_2_ was administered daily via oral gavage at doses of 0 (the control, receiving distilled water), 1, 4, or 10 mg/kg body weight, which dissociates in aqueous solution into [As(OH)_3_], from GD1 to postnatal day 21 (PND21). At the end of the exposure period (PND21), the offspring rats were euthanized via controlled CO_2_ inhalation (30% volume/min displacement rate), ensuring gradual narcosis, followed by immediate cervical dislocation to confirm death. Their whole brains were harvested immediately post-euthanasia. For the molecular analyses (Western blot and qPCR), frozen brains were sectioned in the coronal plane. Then, the hippocampal tissues were carefully dissected along their natural anatomical boundaries under rat brain matrices (ASI Instruments, Houston, TX, USA). For histology (HE/IHC), the brains were fixed in 4% paraformaldehyde (PFA) prior to paraffin embedding and coronal sectioning (4 μm) throughout the hippocampal formation. All experimental procedures were conducted in accordance with the Institutional Animal Care Guidelines of Dalian Medical University and approved by the Animal Ethics Committee of the institution (Permit Number: AEE21048).

### 2.2. Cell Culture and Treatment

Murine microglia cell line BV-2 cells (gifted by Professor Qingshan Wang from Dalian Medical University) were cultured in DMEM (the base medium, Thermo, Waltham, MA, USA) containing 4.5 g/L of D-glucose supplemented with 10% FBS (Thermo), 100 U/mL of penicillin, and 100 μg/mL of streptomycin (HyClone, Logan, UT, USA) at 37 °C under 5% CO_2_. The BV2 microglial cells were seeded into 100 mm culture dishes at a density of 1 × 10^6^ cells/dish in 10 mL of complete growth medium. After 24 h of incubation, the cells were treated with NaAsO_2_ (0, 0.5, and 2 μM) for 24 h. For the experiments involving the inhibitor apocynin (APO, a NOX2 inhibitor; MCE, Parsippany, NJ, USA, 99.93% purity) or MCC950 (full chemical name, a NLRP3 inflammasome inhibitor; MCE, 99.62% purity), the BV2 cells were pretreated with 0.5 mM APO or 100 nM MCC950 for 30 min. Following pretreatment, the supernatant was removed, the cells were washed, and fresh medium containing 2 μM of NaAsO_2_ was added for a further 24 h of incubation. After the respective treatments (NaAsO_2_ alone or NaAsO_2_ following the APO/MCC950 pretreatment), the cell culture supernatant was collected and then centrifuged at 2000× *g* for 5 min to remove cellular debris, generating the conditioned medium (CM). HT22 neuronal cells (gifted by Professor Shengfa Li from Dalian Medical University) were seeded into 100 mm dishes at a density of 5 × 10^5^ cells/dish in 10 mL of complete DMEM. After 24 h, the medium was replaced with the BV2-CM generated from the different treatment groups (NaAsO_2_ alone, APO pretreated+NaAsO_2_, and MCC950 pretreated+NaAsO_2_). The HT22 cells were then exposed to the CM for 48 h prior to the subsequent analysis.

### 2.3. Hematoxylin–Eosin (HE) Staining

HE staining was performed on 4% paraformaldehyde-fixed, paraffin-embedded tissues. Brain tissue sections (of a 4 μm thickness) were deparaffinized in xylene (Fisher Chemical, Fair Lawn, NJ, USA, 2 × 10 min), rehydrated through a graded ethanol series (Sigma-Aldrich, 100%, 95%, 80%, 70%; 2 min each), stained with hematoxylin (MCE, 98.28% purity) for 5 min and eosin (MCE, 99.42% purity) for 1 min, dehydrated in reverse ethanol gradients (70%, 80%, 95%, and 100%; 1 min each), cleared in xylene (2 × 5 min), and finally mounted using a neutral xylene-based mounting medium (Sigma-Aldrich; comprising polystyrene distyrene resin, dibutyl phthalate plasticizer, and xylene solvent). A morphological analysis was conducted under a light microscope (Olympus, Tokyo, Japan).

### 2.4. The Morris Water Maze (MWM) Test

The Morris water maze test (XR business, Shenzhen, Guangdong, China) was employed to assess spatial learning and memory in the offspring rats at PND21. The apparatus consisted of a circular pool (100 cm diameter, 50 cm height) divided into four quadrants (Q1–Q4). A removable platform (of a 10 cm diameter) was submerged 1 cm below the water’s (20–25 °C) surface in Q3. Distinct visual cues were affixed to each quadrant wall. For the positioning navigation test, the offspring rats underwent daily training sessions (3 trials per day, 5 consecutive days). Each trial allowed a maximum swim duration of 90 s; rats failing to locate the platform within this period were gently guided to it and permitted to remain for 5 s, with the escape latency recorded as 90 s. On day 6, the spatial probe test was conducted by removing the platform. The rats swam freely for 90 s while parameters including the escape latency (time to reach the original platform location), target quadrant residence time, and platform area crossings were quantified to evaluate their memory retention.

### 2.5. Western Blot

The HT22 cells and the hippocampal tissue from the offspring rats were lysed in RIPA lysis buffer (comprising 50 mM Tris-HCl pH 7.4, 150 mM NaCl, 1% Triton X-100, 1% sodium deoxycholate, 0.1% SDS; Beyotime, Shanghai, China), supplemented with phenylmethanesulfonyl fluoride (PMSF; Beyotime, ≥98.5% purity) and a broad-spectrum protein phosphatase inhibitor cocktail (Solarbio, Beijing, China). Cultured cells from a single 100 mm dish were lysed in 50 μL of RIPA buffer, whereas 50 mg of the hippocampal tissue sample required 500 μL of RIPA buffer, with lysis conducted for 30 min at 4 °C, involving three 10 s vortex pulses at 10 min intervals. Measurements of the protein concentration were taken using the BCA kit (KeyGen, Nanjing, Jiangsu, China). Protein samples were separated through SDS-PAGE and transferred onto PVDF membranes (Millipore, Temecula, CA, USA). The samples were initially run at a constant voltage (75 V) for approximately 30 min until the protein’s molecular weight markers were fully separated into distinct bands, followed by an increased voltage (120 V) for 60 min, with the total electrophoresis duration adjusted according to the target protein’s molecular weight. After blocking using 5% skimmed milk for 2 h, the membrane was incubated with the primary antibody overnight at 4 °C. The primary antibodies used were as follows: anti-postsynaptic density protein 95 (Beyotime,, 1:1000), anti-synaptophysin (Bioss, Beijing, China; 1:1000), NLRP3 (Wanlei, Shenyang, Liaoning, China; 1:1000), pro-caspase-1 (Affinity, Columbus, Ohio, USA; 1:1000), pro-IL-1β (Wanlei, 1:1000), ASC (Bioss, 1:1000), caspase-1 (Affinity, 1:1000), IL-1β (Wanlei, 1:1000), and anti-β-actin (Proteintech, Rosemont, IL, USA; 1:5000). It was then washed 3 times for 10 min each time using tris-buffered saline with 0.1% Tween-20 (TBST) and incubated with horseradish peroxidase (HRP)-conjugated secondary antibodies (Proteintech, 1:5000) for 2 h at room temperature; washed with TBST 3 times in 30 min; and then photographed using an ECL (KeyGen). The protein expression was evaluated using Image J software (version 1.54p).

### 2.6. Immunofluorescence Staining

The brain sections were subjected to a standardized deparaffinization and rehydration protocol. Briefly, the tissues underwent sequential immersion in three changes of fresh 100% xylene (5 min each), followed by dehydration in graded ethanol solutions: two changes of 100% ethanol, 95% ethanol, 90% ethanol, and 75% ethanol (5 min each). The sections were then rinsed in triple-distilled water A and B (5 min each) to complete the rehydration. Antigen retrieval was performed using preheated Citrate Antigen Retrieval Solution (10 mM sodium citrate, 0.05% Tween 20, pH 6.0; Beyotime) to optimize the epitope exposure. After cooling to room temperature, nonspecific binding sites were blocked using 5% bovine serum albumin (BSA) for 1 h at 25 °C. The sections were incubated overnight at 4 °C with a rabbit polyclonal primary antibody against Iba-1 (Wanlei, 1:100). Following the three washes, the sections were incubated with goat anti-rabbit Alexa Fluor 594 (Abcam, Cambridge, MA, USA, 1:500) for 2 h. Nuclei were counterstained using an antifade mounting medium containing 1.5 μg/mL of 4′,6-diamidino-2-phenylindole (Vector Labs, Newark, CA, USA). Fluorescent signals were visualized and captured using a fluorescence microscope (Olympus).

### 2.7. Real-Time qPCR

Total RNA was isolated from both the rat hippocampal tissues and the BV2 cells using TRIzol reagent (Invitrogen, Carlsbad, CA, USA) according to the manufacturer’s instructions. RNA (1 μg) was reverse-transcribed into cDNA using the PrimeScript RT Reagent Kit (Takara, Beijing, China) with oligo (dT) primers in a 20 μL reaction volume. Quantitative real-time PCR was performed using SYBR Green Pro Taq HS Premix (Accurate Biology, Changsha, Liaoning, China) on a QuantStudio 3 system (Applied Biosystems, Foster City, CA, USA). All primers were commercially synthesized (HPLC-purified, Sangon Biotech, Shanghai, China). Each 20 μL reaction contained 10 μL of 2 × SYBR Premix, 0.8 μL of the forward primer (10 μM), 0.8 μL of the reverse primer (10 μM), 2 μL of the cDNA template (diluted 1:10), and 6.4 μL of nuclease-free water. The selected primers are listed in Table 1. The thermal cycling conditions comprised initial denaturation at 95 °C for 30 s and 40 cycles of 95 °C for 5 s (denaturation), 60 °C for 30 s (annealing/extension), and 60 °C to 95 °C (0.5 °C/s increment, melt curve analysis). GAPDH served as the endogenous control. The relative gene expression was calculated using the 2^−ΔΔCt^ method.

### 2.8. NOX2 siRNA Transfection

NOX2 siRNAs and non-targeting control siRNA (GenePharma, Shanghai, China) were transfected into the BV2 cells. First, the BV2 cells were seeded at 1 × 10^5^ cells/well into 6-well plates in 2 mL of complete medium and cultured for 24 h. For transfection, two separate mixtures were prepared in 1.5 mL tubes: Tube A contained 125 μL of Opti-MEM (a reduced-serum medium optimized for transfection, Thermo Fisher, Waltham, MA, USA) mixed with 3.5 μL of 20 μM NOX2 siRNA, while Tube B contained 125 μL of Opti-MEM mixed with 3.5 μL of Lipofectamine 3000 (a proprietary, ready-to-use cationic lipid transfection reagent, Thermo Fisher), prepared according to the manufacturer’s instructions. Following separate 5 min incubations at room temperature, the solutions were combined and gently mixed and then incubated for 20 min at room temperature to form complexes. The resultant mixture was added dropwise to each well, and the cells were incubated with the complexes for 6 h. Subsequently, the cells were exposed to 2 μM of NaAsO_2_ for 24 h prior to the analysis.

### 2.9. Statistical Analysis

The statistical analyses were performed using SPSS 26.0 software. Data normality was assessed using the Shapiro–Wilk test. Normally distributed data were analyzed using a one-way ANOVA with Tukey’s post hoc test for multiple comparisons. Non-normally distributed variables were subjected to Kruskal–Wallis tests followed by Dunn’s post hoc correction. Statistical significance was defined as *p* < 0.05.

## 3. Results

### 3.1. Gestational/Lactational NaAsO_2_ Exposure Induces Learning and Memory Impairments in Offspring Rats

**Figure 1:**
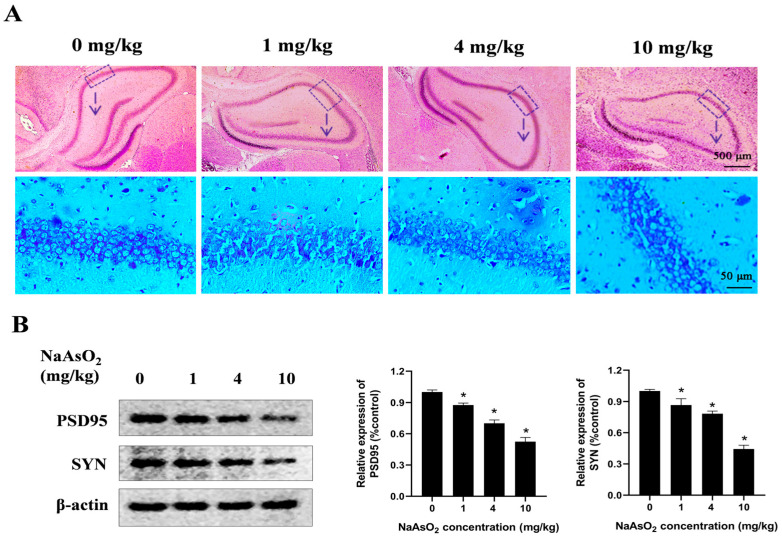
Gestational/lactational NaAsO_2_ exposure induces hippocampal tissue damage in offspring rats. SD rats were gavaged with NaAsO_2_ (0, 1, 4, and 10 mg/kg body weight) from gestational day 1 to postnatal day 21 (PND21). Offspring was sacrificed on PND21. (**A**) Pathological changes in the hippocampus were observed through HE staining (scale bar = 500 μm, 4× magnification; scale bar = 50 μm, 20× magnification; arrow, the dashed box area enlarged below). (**B**) PSD95 and SYN in the hippocampus were detected using a Western blot analysis. Full Western blot images can be found in Figure S1. The data are expressed as mean ± standard error (SEM). n = 3–12 in each group; * *p* < 0.05 vs. control.

To determine whether developmental NaAsO_2_ exposure induces learning and memory impairments in offspring, a rat model was generated through oral gavage administration of NaAsO_2_ at doses of 0, 1, 4, and 10 mg/kg NaAsO_2_ from GD1 to PND21. The histological analysis (Figure 1A) revealed mild disruption in the hippocampal architecture in the 4 mg/kg NaAsO_2_ group, while the 10 mg/kg group exhibited pronounced histopathological damage, characterized by loosely arranged pyramidal cell layers, a vacuolated neuropil, and a disordered distribution of glial cells, showing marked differences compared to the controls. PSD95 and SYN play important roles in learning and memory [29,30,31]. As shown in Figure 1B, compared with the control group, the levels of PSD95 and SYN proteins decreased after NaAsO_2_ exposure in a dose-dependent manner.

**Figure 2:**
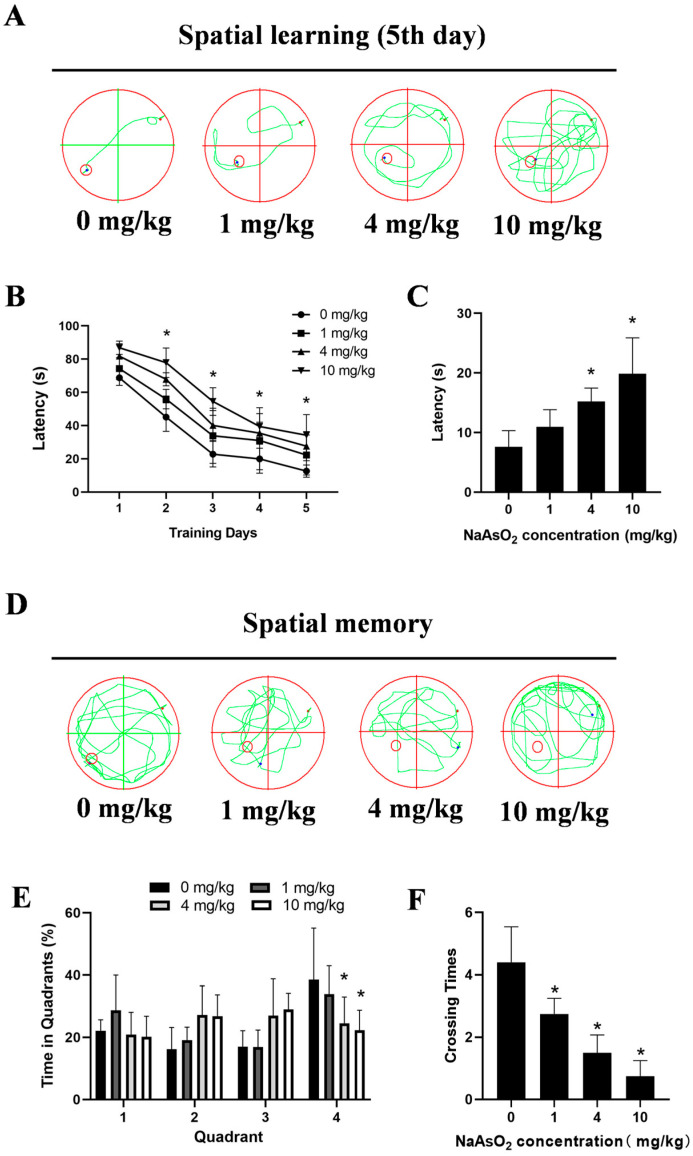
Gestational/lactational NaAsO_2_ exposure induces learning–memory deficits in offspring rats. SD rats were gavaged with NaAsO_2_ (0, 1, 4, and 10 mg/kg body weight) from gestational day 1 to postnatal day 21 (PND 21). PND21 rats were subjected to the Morris water maze test. (**A**) Day 5 learning path panel. Small red circle, escape platform location; small red dot, rat entry point; small blue dot, rat exit point. (**B**) Escape latency in the first 5 days. (**C**) Escape latency in the probe test. (**D**) Probe trial path panel. Small red circle, escape platform location; small red dot, rat entry point; small blue dot, rat exit point. (**E**) Quadrant times in the probe test. (**F**) Crossings to the original platform location. Data are expressed as the mean ± SEM. n = 10–12 in each group; * *p* < 0.05 vs. control.

The MWM tests showed impaired learning, indicated by significantly longer latencies to locate the hidden platform on day 5 (7.61 ± 1.21 s, 10.95 ± 1.44 s, 15.21 ± 1.13 s, and 19.86 ± 3.01 s for the 0, 1, 4, and 10 mg/kg groups, respectively) in the NaAsO_2_-exposed offspring (Figure 2A,B). Spatial probe tests indicated memory deficits, with the NaAsO_2_ groups taking longer to reach the original location of the platform (Figure 2C), spending less time in the original target quadrant (Figure 2D,E), and showing fewer crossings to the original platform location (Figure 2F). These data collectively indicate developmental NaAsO_2_-exposure-induced hippocampal damage and learning–memory deficits.

### 3.2. Gestational/Lactational NaAsO_2_ Exposure Causes Microglial Activation in the Hippocampus of Offspring Rats

**Figure 3:**
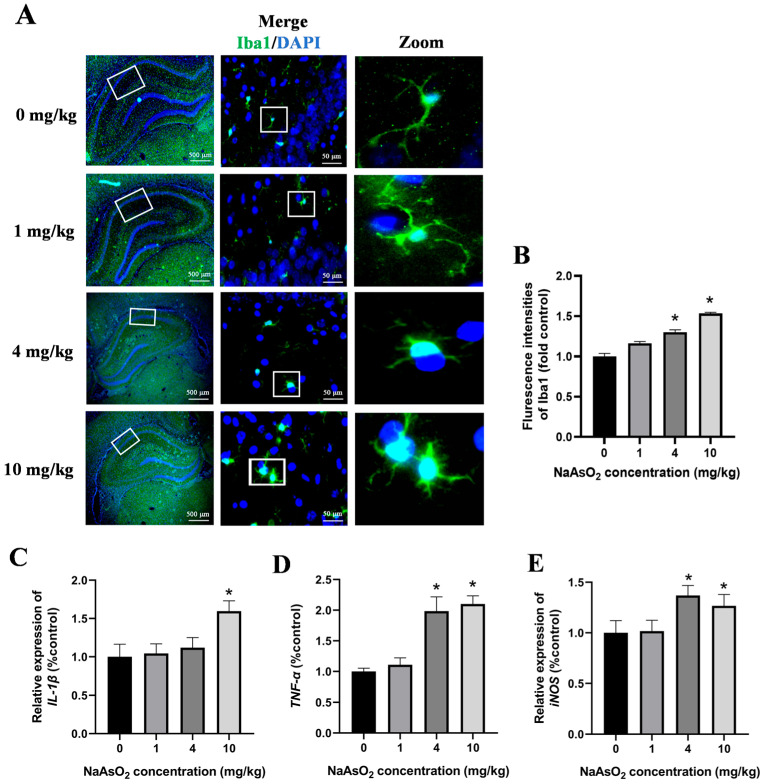
Gestational/lactational NaAsO_2_ exposure causes microglial activation in the hippocampus of offspring rats. (**A**) Immunofluorescence staining using the anti-Iba-1 antibody was performed, and representative images are shown (scale bar = 500 μm, 4× magnification; scale bar = 50 μm, 20× magnification; white box, the area magnified in the right panel.). (**B**) The density of green fluorescence of Iba1 was quantified. The (**C**) IL-1β, (**D**) TNF-α, and (**E**) iNOS gene expression in the hippocampus was measured through real-time PCR. Data are expressed as the mean ± SEM. n = 3–6 in each group; * *p* < 0.05 vs. control.

The activation of the microglia in the hippocampal tissues of the offspring rats was assessed through immunostaining for Iba-1, a specific marker of the microglia. As illustrated in Figure 3A,B, the NaAsO_2_-exposed groups exhibited a significantly enhanced Iba-1 fluorescence intensity and enlarged fluorescence areas in the hippocampus compared to these values in the control group. A morphological analysis further revealed marked hypertrophy of the microglial cells, characterized by thickened and enlarged cellular processes, with these changes becoming progressively more pronounced as the NaAsO_2_ exposure dose increased, indicating dose-dependent microglial activation following prenatal and lactational NaAsO_2_ exposure. To investigate the impact of NaAsO_2_ exposure on the expression of microglial-activation-associated genes in the hippocampal tissue, mRNA levels of IL-1β, TNF-α, and iNOS were quantified using real-time PCR. The results depicted in Figure 3C–E showed a dose-dependent increase in the expression of all genes, suggesting that NaAsO_2_ exposure during the developmental periods activated the microglia in the hippocampal tissues.

### 3.3. The NLRP3 Inflammasome Contributes to NaAsO_2_-Induced Activation of the Microglia

**Figure 4:**
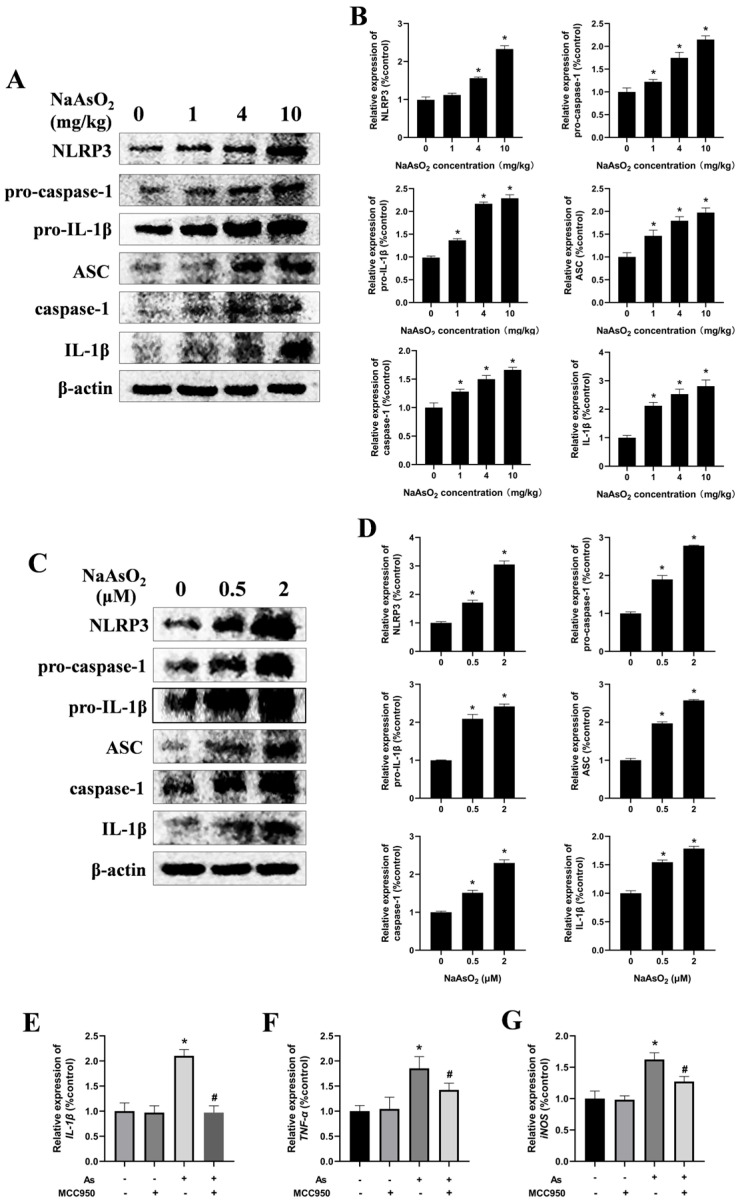
The NLRP3 inflammasome contributes to NaAsO_2_-induced activation of the microglia. (**A**) NLRP3, pro-caspase-1, pro-IL-1β, ASC, caspase-1, and mature IL-1β in the hippocampus of NaAsO_2_-exposed offspring rats were detected through a Western blot analysis, and (**B**) the density of the blots was quantified. Full Western blot images can be found in Figure S2. (**C**) BV2 microglial cells were treated with 0, 0.5, and 2 μM of NaAsO_2_. After 24 h, the expression of NLRP3, pro-caspase-1, pro-IL-1β, ASC, caspase-1, and mature IL-1β was detected through a Western blot analysis, and (**D**) the density of the blots was quantified. Full Western blot images can be found in Figure S3. (**E**) BV2 microglial cells were treated with 2 μM of NaAsO_2_ with or without MCC950 (an inhibitor of the NLRP3 inflammasome). After 24 h, the gene expression of (**E**) IL-1β, (**F**) TNF-α, and (**G**) iNOS was measured through real-time PCR. Data are expressed as the mean ± SEM. n = 3–6 in each group; * *p* < 0.05 vs. control, ^#^ *p* < 0.05 vs. the As group.

The NLRP3 inflammasome, a protein complex comprising NLRP3, ASC, and caspase-1, plays a critical role in mediating inflammatory responses by promoting the release of cytokines such as TNF-α and IL-1β [18,32]. To investigate whether the NLRP3 inflammasome is involved in microglial activation, NLRP3-inflammasome-related proteins in the hippocampal tissues of the offspring rats were analyzed via Western blot (Figure 4A,B). The results demonstrated a dose-dependent increase in the protein levels of NLRP3, pro-caspase-1, pro-IL-1β, ASC, caspase-1, and mature IL-1β, indicating NaAsO_2_-induced activation of the NLRP3 inflammasome in vivo. For in vitro validation, BV-2 cells—a well-established murine microglial cell line that retains the morphological, phenotypic, and functional characteristics of the primary microglia [33,34]—were exposed to NaAsO_2_ for 24 h. Consistent with the in vivo results, NaAsO_2_ exposure induced marked upregulation of the NLRP3, pro-caspase-1, pro-IL-1β, ASC, caspase-1, and mature IL-1β proteins in the NaAsO_2_-treated cells (Figure 4C,D).

To delineate the role of the NLRP3 inflammasome in NaAsO_2_-induced microglial activation further, the BV-2 cells were pretreated with 100 nM of MCC950, a selective NLRP3 inflammasome inhibitor, for 30 min prior to NaAsO_2_ exposure. The real-time PCR results demonstrated that the MCC950 pretreatment markedly suppressed the expression of microglial-activation-associated genes, including IL-1β, TNF-α, and iNOS (Figure 4E–G). These collective findings indicate that the NLRP3 inflammasome contributes to NaAsO_2_-induced activation of the microglia.

### 3.4. NOX2 Is Key to NLRP3 Inflammasome Activation in NaAsO_2_-Exposed Microglia

**Figure 5:**
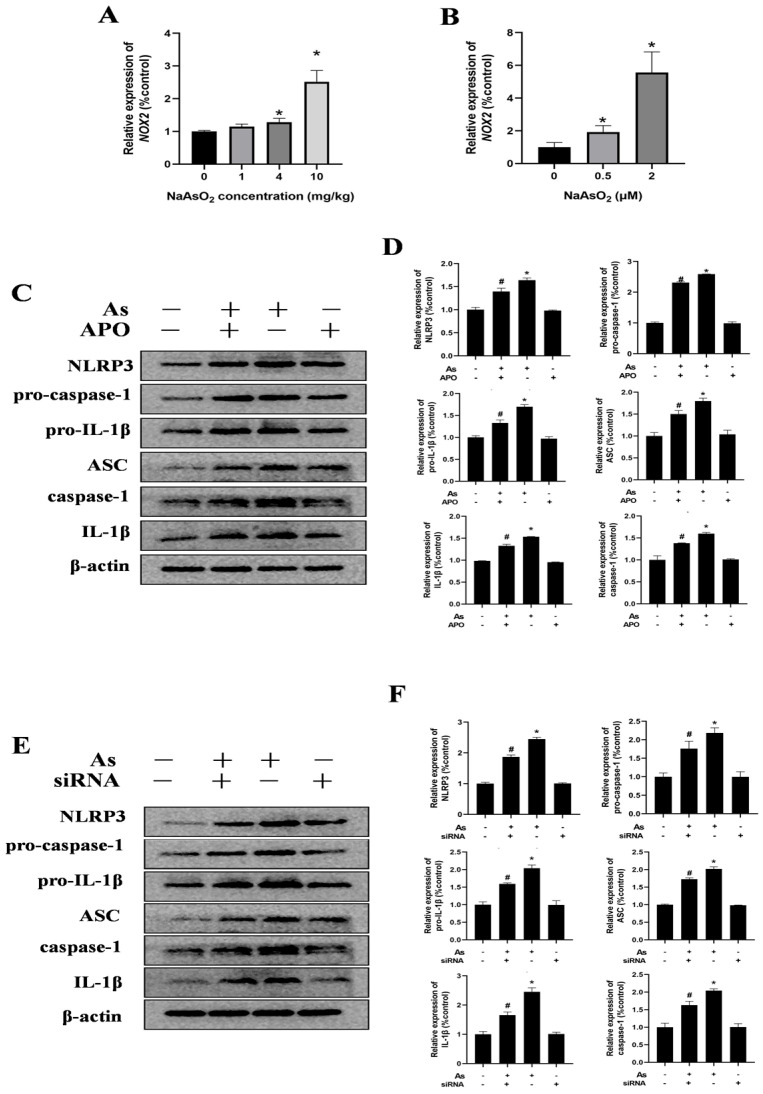
NOX2 is key to NLRP3 inflammasome activation in NaAsO_2_-exposed microglia. The NOX2 gene expression in the hippocampus (**A**) and BV2 microglial cells (**B**) was measured through real-time PCR. (**C**) BV2 microglial cells were treated with 2 μM of NaAsO_2_ with or without APO pretreatment (a NOX2 inhibitor). After 24 h, the expression of NLRP3, pro-caspase-1, pro-IL-1β, ASC, caspase-1, and mature IL-1β was detected through a Western blot analysis, and (**D**) the density of the blots was quantified. Full Western blot images can be found in Figure S4. (**E**) BV2 microglial cells were treated with 2 μM of NaAsO_2_ with or without siNOX2. After 24 h, the expression of NLRP3, pro-caspase-1, pro-IL-1β, ASC, caspase-1, and mature IL-1β was detected through a Western blot analysis, and (**F**) the density of the blots was quantified. Full Western blot images can be found in Figure S5. Data are expressed as the mean ± SEM. n = 3–6 in each group; * *p* < 0.05 vs. Control, ^#^ *p* < 0.05 vs. the As group.

NADPH oxidases (NOX), particularly NOX2 (also known as gp91phox), are the predominant isoform in the hippocampal tissues and microglia. To assess the impact of gestational/lactational NaAsO_2_ exposure on the NOX2 expression in the offspring, real-time PCR was performed. As shown in Figure 5A, NaAsO_2_ exposure induced dose-dependent upregulation of the NOX2 mRNA levels compared to those in the control group. Similarly, increased NOX2 gene levels were observed in the NaAsO_2_-exposed BV-2 cells in a dose-dependent manner (Figure 5B). To investigate the functional link between NOX2 and NLRP3 inflammasome activation in NaAsO_2_-induced microglial activation, we pretreated the BV-2 cells with 0.5 mM of APO (a NOX2 inhibitor) for 30 min prior to NaAsO_2_ (2 μM) exposure. The Western blot analysis (Figure 5C,D) revealed that the APO pretreatment significantly attenuated the NaAsO_2_-induced upregulation of the NLRP3 inflammasome components, including NLRP3, pro-caspase-1, pro-IL-1β, ASC, cleaved caspase-1, and mature IL-1β proteins. We further examined NOX2’s specific role using NOX2-targeted siRNA (siNOX2) in the BV-2 cells. The Western blot results (Figure 5E) demonstrated that NOX2 knockdown markedly suppressed the NaAsO_2_-induced upregulation of the NLRP3, pro-caspase-1, pro-IL-1β, ASC, cleaved caspase-1, and mature IL-1β proteins. These findings confirm that NOX2 is essential for NaAsO_2_-induced NLRP3 inflammasome activation in the microglia.

### 3.5. Inhibition of the Microglial NLRP3 Inflammasome or NOX2 Mitigates NaAsO_2_-Induced Impairment in Hippocampal HT22 Cells

**Figure 6:**
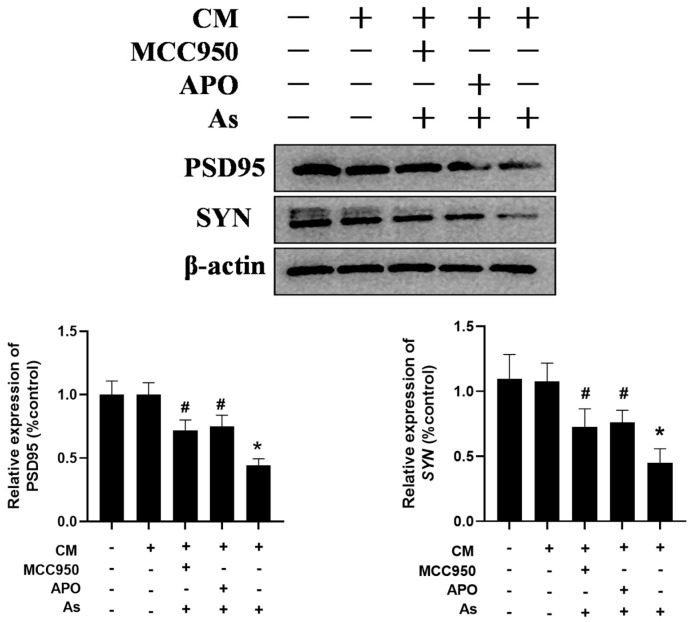
Inhibition of the microglial NLRP3 inflammasome or NOX2 mitigates NaAsO_2_-induced impairments in hippocampal HT22 cells. BV2 microglial cells were treated with 2 μM of NaAsO_2_ with or without MCC950 or APO pretreatment. After 24 h, the conditioned medium (CM) was collected and then added to the hippocampal HT22 cells. The PSD95 and SYN levels in the HT22 cells, important mediators of learning and memory, were measured using a Western blot analysis after 48 h of CM treatment. Full Western blot images can be found in Figure S6. Data are expressed as the mean ±S EM. n = 3–4 in each group; * *p* < 0.05 vs. control, ^#^ *p* < 0.05 vs. the As group.

To confirm the role of the microglial NLRP3 inflammasome and NOX2 in NaAsO_2_-induced impairments in the hippocampal neurons, the BV2 microglial cells were pretreated with either 0.5 mM of APO or 100 nM of MCC950 for 30 min. The supernatant was collected and then administered to the hippocampal HT22 cells in vitro. The HT22 cell line, derived from hippocampal neurons from mice, retains the typical characteristics of the hippocampal neurons and is commonly used as a model of the hippocampal neurons. As shown in Figure 6, the conditioned medium (CM) prepared from the NaAsO_2_-treated BV2 microglial cells decreased the PSD95 and SYN expression, important mediators of learning and memory, in the HT22 cells. Meanwhile, the disruption of PSD95 and SYN were marked as reversed in the HT22 cells treated with the CM derived from the combined NaAsO_2_- and MCC950/APO-treated microglia.

## 4. Discussion

In the present study, we demonstrated that NOX2-mediated microglial NLRP3 inflammasome activation might contribute to learning and memory impairments in the offspring of rats exposed to As(III) at gestation/lactation. Salient features of our study are that (1) gestational/lactational As(III) exposure preceded the learning and memory impairments in the offspring rats; (2) As(III) exposure activated the microglia in the hippocampus of the offspring; (3) the NLRP3 inflammasome mediated microglial activation; and (4) NOX2 was involved in NLRP3 inflammasome activation. Pharmacological inhibition of NOX2 or the NLRP3 inflammasome protected the hippocampal HT22 cells against neurotoxicity.

Inorganic arsenic [As(III) and As(V)] is a ubiquitous environmental pollutant which primarily enters the human body through contaminated drinking water. Chronic inorganic arsenic exposure, particularly maternal exposure, causes irreversible cognitive deficits. Emerging evidence suggests that arsenite-induced neurodevelopmental damage is closely associated with microglial activation [12,13,35,36,37]. At present, many hypotheses, such as reactive oxygen species [38], the IRE1/XBP axis [39], and ATP-binding cassette transporters [40], have been proposed to explain the pathogenesis of microglial activation. Further research is still needed to elucidate the exact mechanism.

In this study, we demonstrated that the NLRP3 inflammasome plays a crucial role in the microglial activation and neurotoxicity induced by gestational/lactational arsenite exposure in rat offspring. The NLRP3 inflammasome is an intracellular signaling complex highly expressed in the microglia. As(III) treatment induced microglial NLRP3 inflammasome activation in both the hippocampal tissues in vivo and microglial BV2 cells in vitro, as evidenced by increased NLRP3 and ASC expression, caspase-1 activation, and IL-1β production. Moreover, pharmacological inhibition of the NLRP3 inflammasome significantly mitigated the As(III)-induced microglial activation and neuronal damage, confirming its central role. Notably, our findings align with the mechanisms established for other prevalent environmental metals. Similar to As(III), Cd(II) [41,42,43], Pb(II) [25,44,45], and Mn(II) [21,46,47] activate the NLRP3 inflammasome both in vivo and in vitro. For instance, Cd(II) exposure (1 mg/kg CdCl_2_, 8 weeks of intraperitoneal injection) induces neuronal injury via NLRP3 activation, which is reversible using the MCC950 inhibitor [42]. Chronic Pb(II) exposure (100 ppm of Pb(CH_3_COOH)_2_ in drinking water for 10 weeks) triggers NLRP3-dependent microglial activation and cognitive deficits that are ameliorated by NLRP3 ablation [25]. Similarly, Mn(II) treatment (30 mg/kg MnCl_2_, intranasally administered for 21 days) impairs learning/memory via NLRP3 inflammasome activation [21]. These collective observations establish the NLRP3 inflammasome as a convergent signaling hub for diverse neurotoxic metals.

Microglial NOX2, which generates superoxide, is involved in regulating NLRP3 inflammasome activation [48,49,50]. This is evidenced by the suppressed NLRP3 activation in NOX2-deficient models and p47phox (a cytosolic NOX2 subunit) knockout cells [48,49], as well as the pharmacological inhibition of NLRP3 via NOX2 downregulation using NSAIDs-Se derivatives [50]. Specifically, we found that As(III) upregulated the NOX2 expression in both the hippocampal tissues in vivo and the BV2 cells in vitro. Crucially, pharmacological or genetic inhibition of NOX2 significantly attenuated NLRP3 inflammasome activation in the As(III)-exposed microglia. Importantly, the neuronal damage mediated by the As(III)-activated microglia was substantially rescued by blocking NOX2 or through NLRP3 blockade, thus establishing a functional As(III)-NOX2-NLRP3–neurotoxicity axis in the microglia. These results bear significant public health implications. Considering the frequent co-occurrence of multiple neurotoxic metals in groundwater contamination—exemplified by arsenic/manganese in Bangladeshi tubewells and arsenic/lead in mining regions [51,52]—our findings establish the NOX/NLRP3 axis as a unified therapeutic target for mixed-metal neurotoxicity. Future research should prioritize investigating the synergistic interactions between metal combinations (e.g., As(III) + Pb(II) or Mn(II)) in NOX/NLRP3 pathway activation.

Nevertheless, the precise mechanisms driving As(III)-induced microglial NOX2 activation remain incompletely characterized. Previous work has established PKCδ as a critical regulator of p47phox activation in methamphetamine-exposed microglia, a process demonstrating significant glutathione (GSH) dependence [53]. Notably, As(III) avidly complexes with thiol groups, including GSH, leading to GSH depletion [54]. This context highlights the importance of the findings of Gailer et al., which demonstrated that in vivo co-exposure to As(III) and selenite [Se(IV)] at an 8:1 molar ratio generates a unique (GS)AsSe metabolite [55,56]. This suggests that under selenium co-exposure conditions, additional GSH-depleting pathways may operate through arsenic–selenium complexation. In our model, which involves no exogenous selenium, we hypothesize that PKCδ activation and direct As(III)-GSH complexation deplete GSH reserves, thereby regulating NOX2 activation. This process may subsequently activate the NLRP3 inflammasome and contribute to neurotoxicity. Therefore, future work should not only elucidate the roles of PKCδ and GSH depletion in As(III)-induced microglial NOX2 activation but also examine the potential interactions with selenium where applicable, with careful attention to selenite’s stoichiometry.

In summary, our study demonstrates that microglial NLRP3 inflammasome activation in a NOX2-dependent manner contributes to As(III)-induced neurodevelopmental deficits, particularly in learning and memory impairments. These findings uncover a novel mechanism of As(III)-induced neurodevelopmental toxicity based on new insight into NOX2-dependent NLRP3 inflammasome activation.

## 5. Conclusions

Developmental As(III) exposure induces cognitive deficits in offspring rats by activating the NLRP3 inflammasome in the hippocampal microglia. We demonstrate that As(III) triggers NOX2-dependent NLRP3 inflammasome signaling, evidenced by the upregulated NLRP3, ASC, caspase-1, and IL-1β expression in vivo and in vitro. Pharmacological inhibition of NOX2 (APO) or NLRP3 (MCC950) attenuated the microglial activation and subsequent neuronal damage in the hippocampal HT22 cells. These findings establish the NOX2/NLRP3 axis as a central pathway in As(III)-induced neurodevelopmental toxicity, revealing novel mechanistic targets for preventing environmental arsenic neurotoxicity. Future studies should investigate this pathway in the context of mixed-metal neurotoxicity relevant to groundwater contamination.

## Figures and Tables

**Table 1:** Real-time qPCR primer sequences.

	Forward Primer	Reverse Primer
IL-1β	CCCAAGCACCTTCTTTTCCTT	TCAGACAGCACGAGGCATTT
TNF-α	CAAGAGCCCTTGCCCTAAGG	GGACTCCGTGATGTCTAAGTACTT
iNOS	CCTCACCTACTTCCTGGACATCA	GGGTTGTTGCTGAACTTCCAA
NOX2	GACCTCTGCCCCTGAGGAAA	CCAAAGGGCCCATCAACTG
GAPDH	ATGCCGCCTGGAGAAACC	GCATCAAAGGTGGAAGAATGG

## Data Availability

The data presented in this study are available upon request from the corresponding author.

## Supplementary Materials

The following supporting information can be downloaded at https://www.mdpi.com/article/10.3390/toxics13070538/s1. Figure S1. Full Western blot images for Figure 1B; Figure S2. Full Western blot images for Figure 4A; Figure S3. Full Western blot images for Figure 4C; Figure S4. Full Western blot images for Figure 5A; Figure S5. Full Western blot images for Figure 5E; Figure S6. Full Western blot images for Figure 6.
