# Supplementary material for: NOX2/NLRP3-Inflammasome-Dependent Microglia Activation Promotes As(III)-Induced Learning and Memory Impairments in Developmental Rats
Source: Toxics. 2025 Jun 26;13(7):538. doi: 10.3390/toxics13070538 (PMC12299122; doi:10.3390/toxics13070538)
Supplement: Supplementary file 1 [file toxics-13-00538-s001.zip › toxics-3618354-supplementary.pdf]

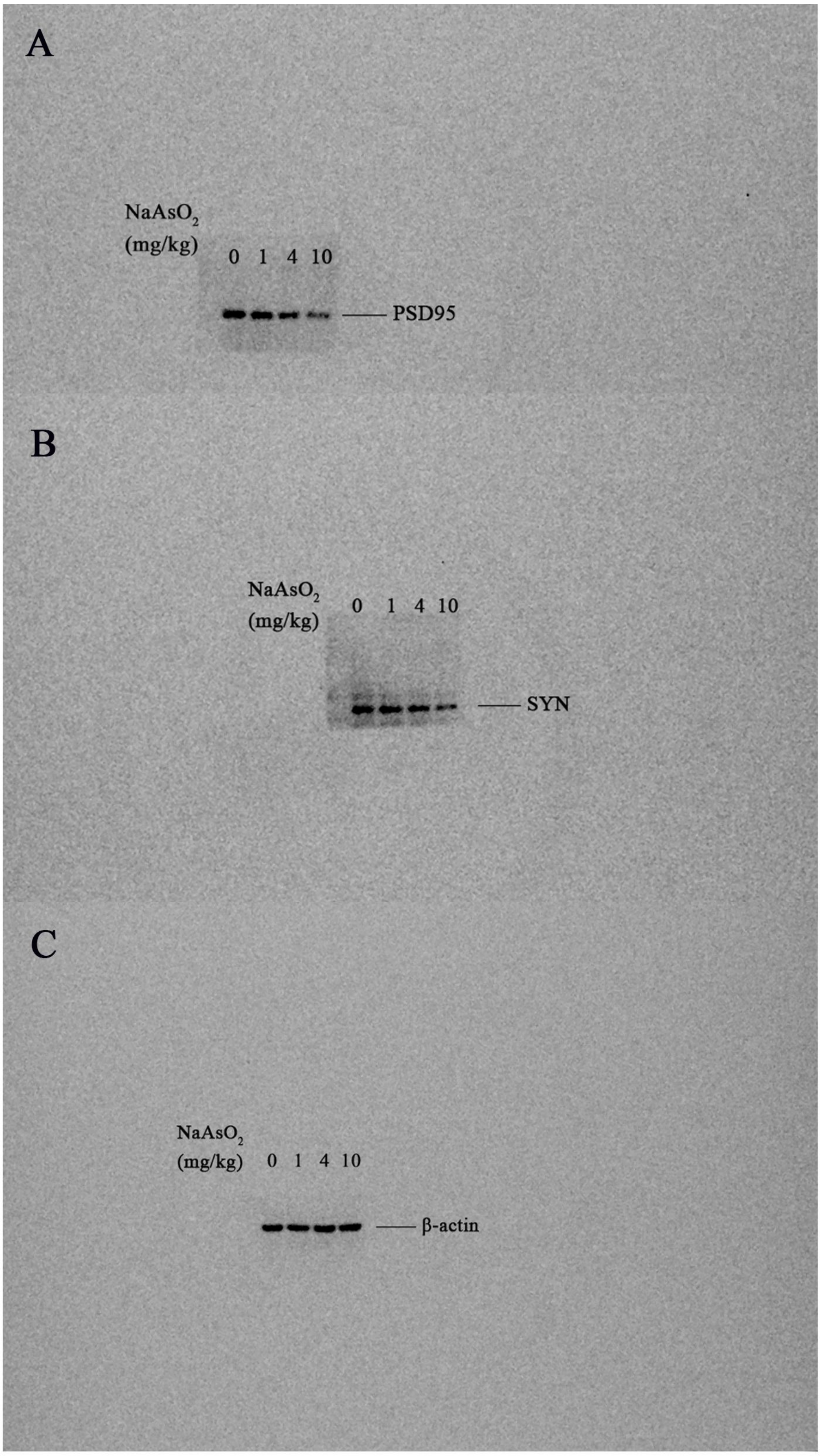

Figure S1. Full western blot images for Figure 1B.

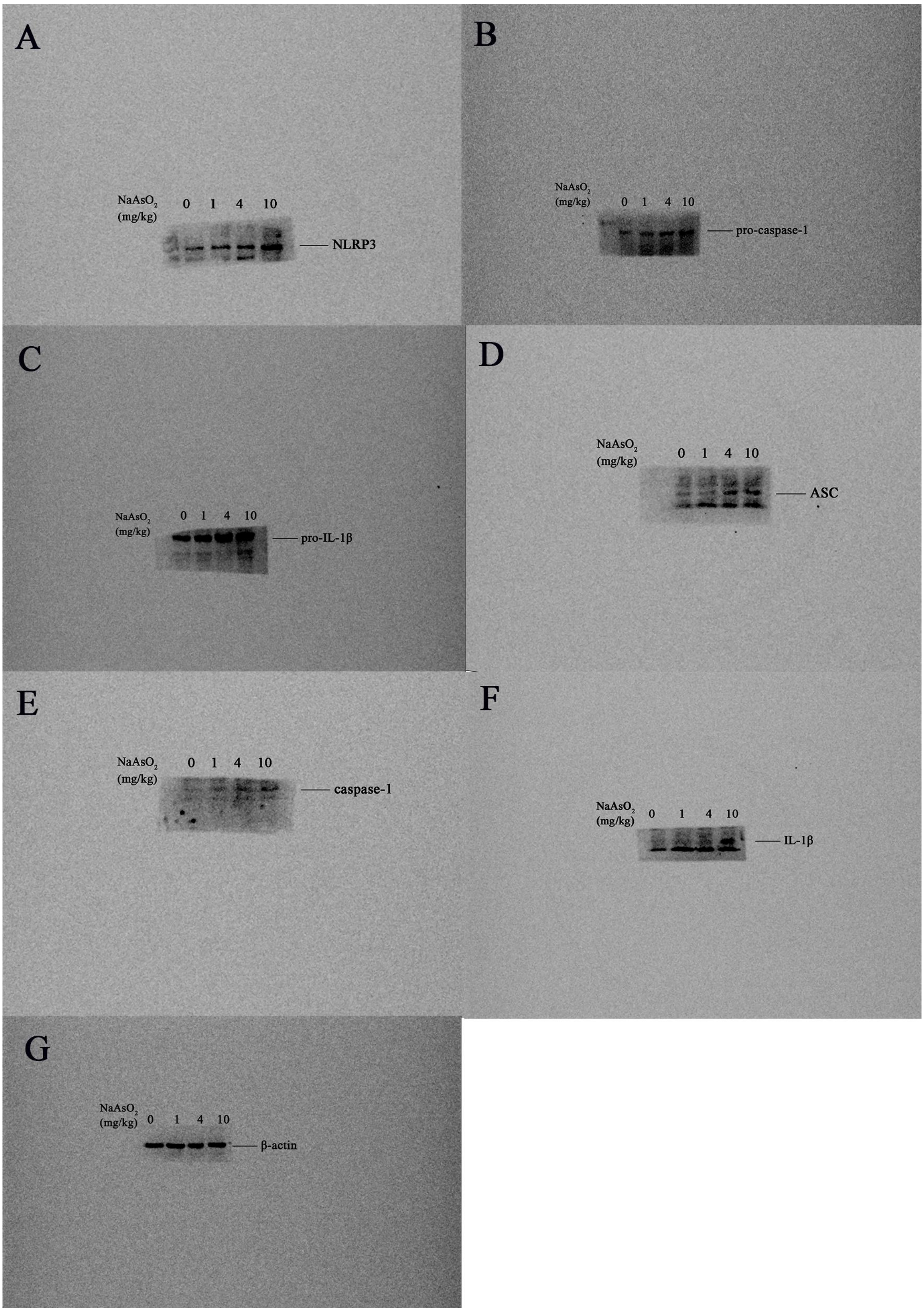

Figure S2. Full western blot images for Figure 4A.

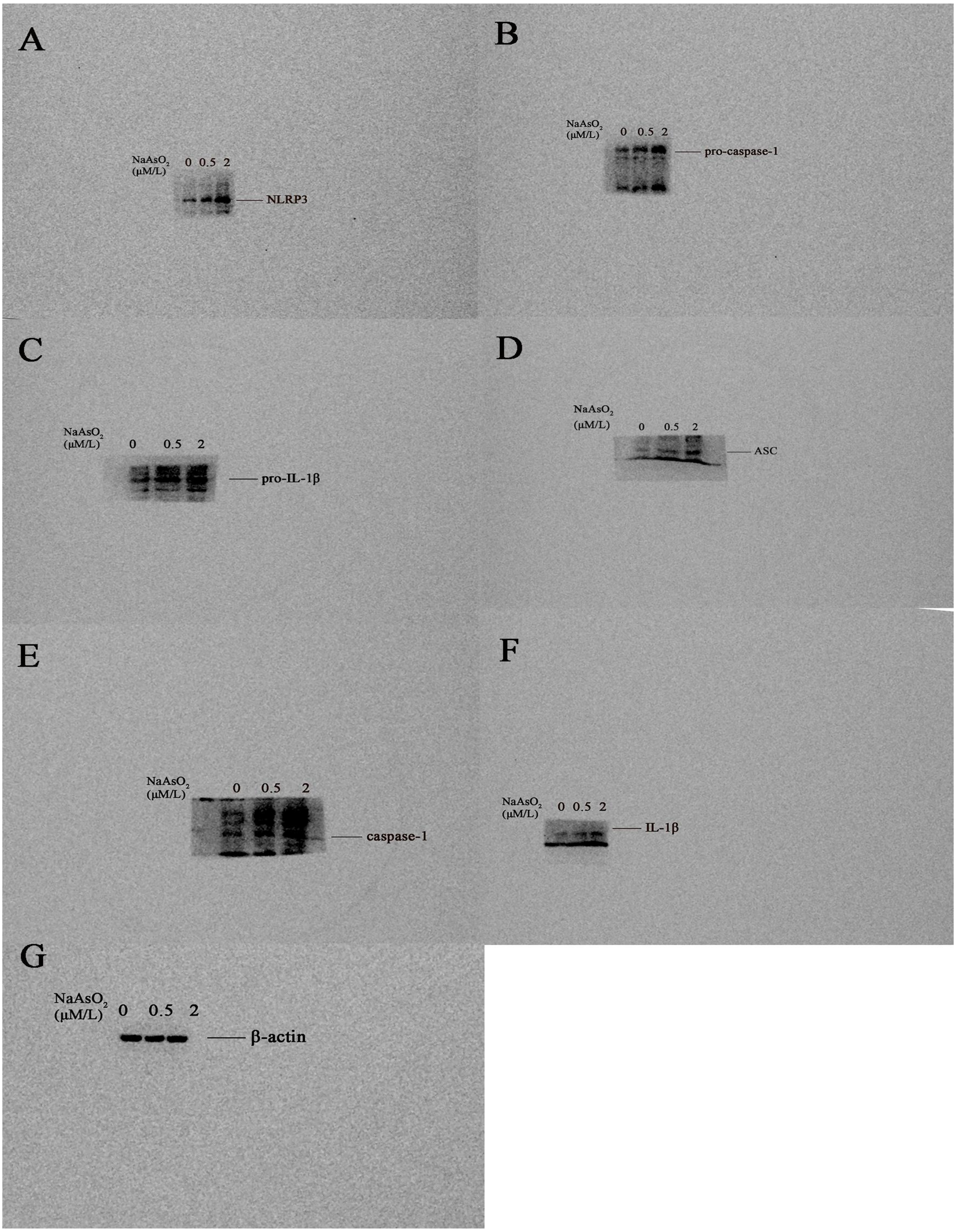

Figure S3. Full western blot images for Figure 4C.

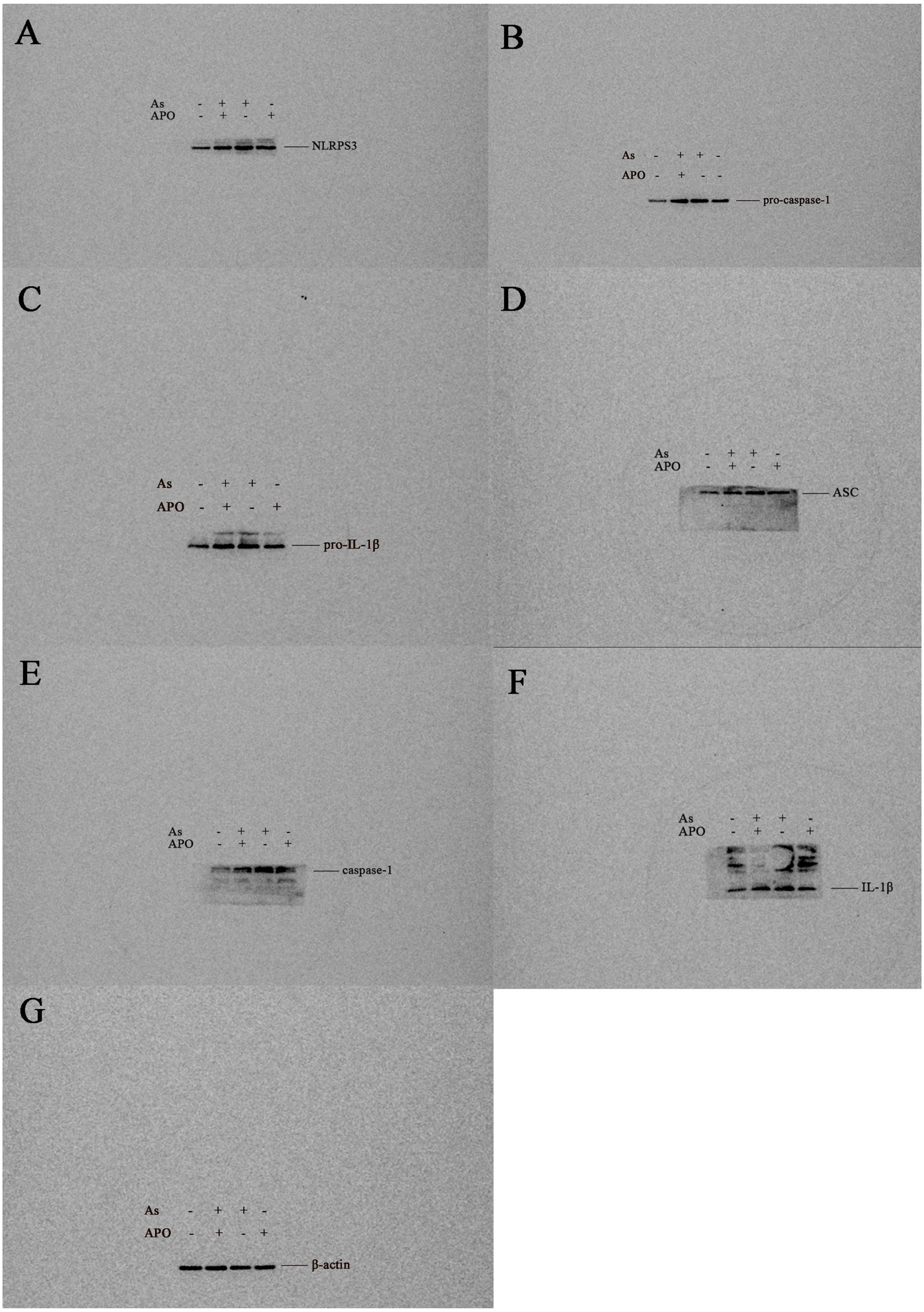

Figure S4. Full western blot images for Figure 5C.

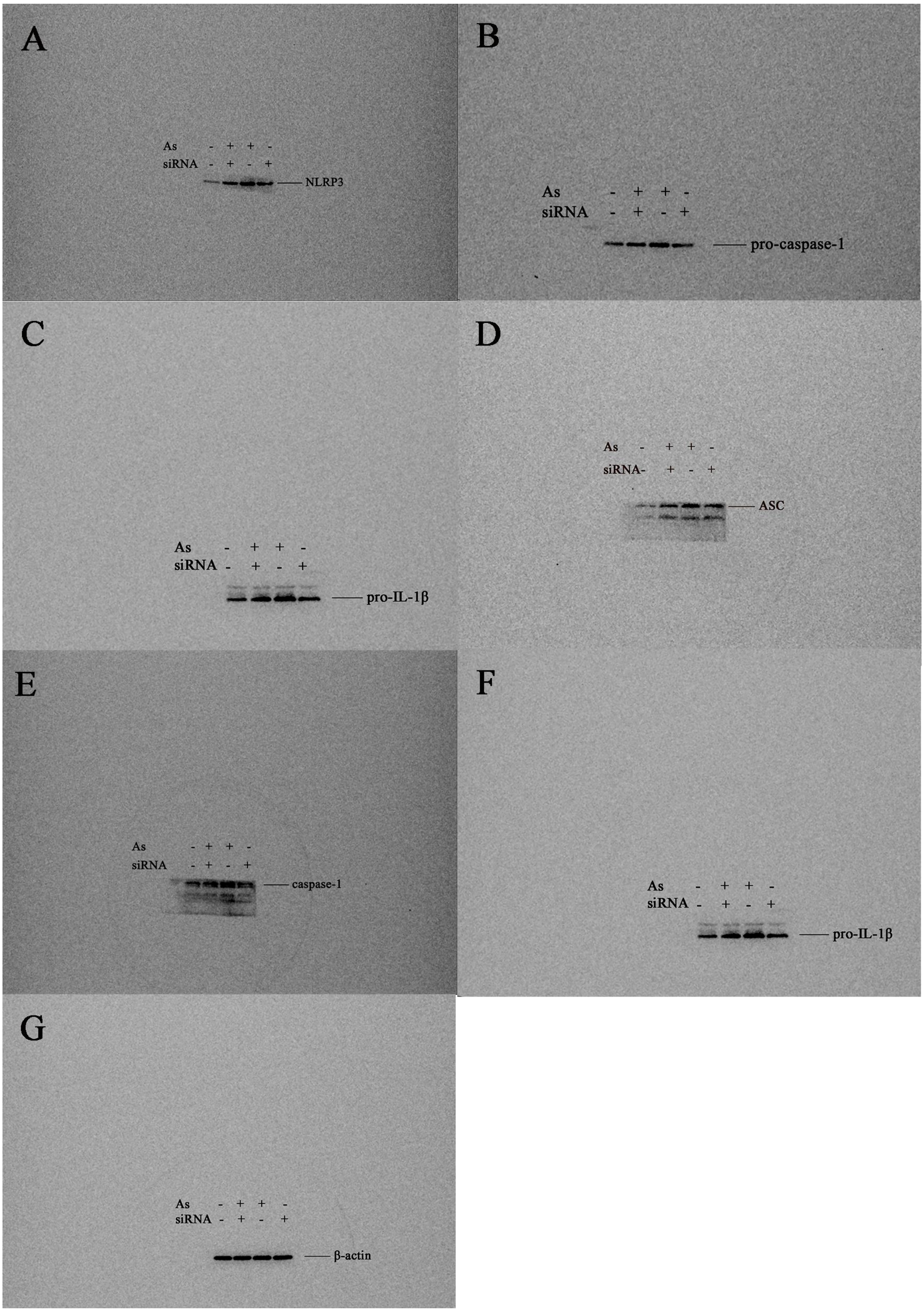

Figure S5. Full western blot images for Figure 5E.

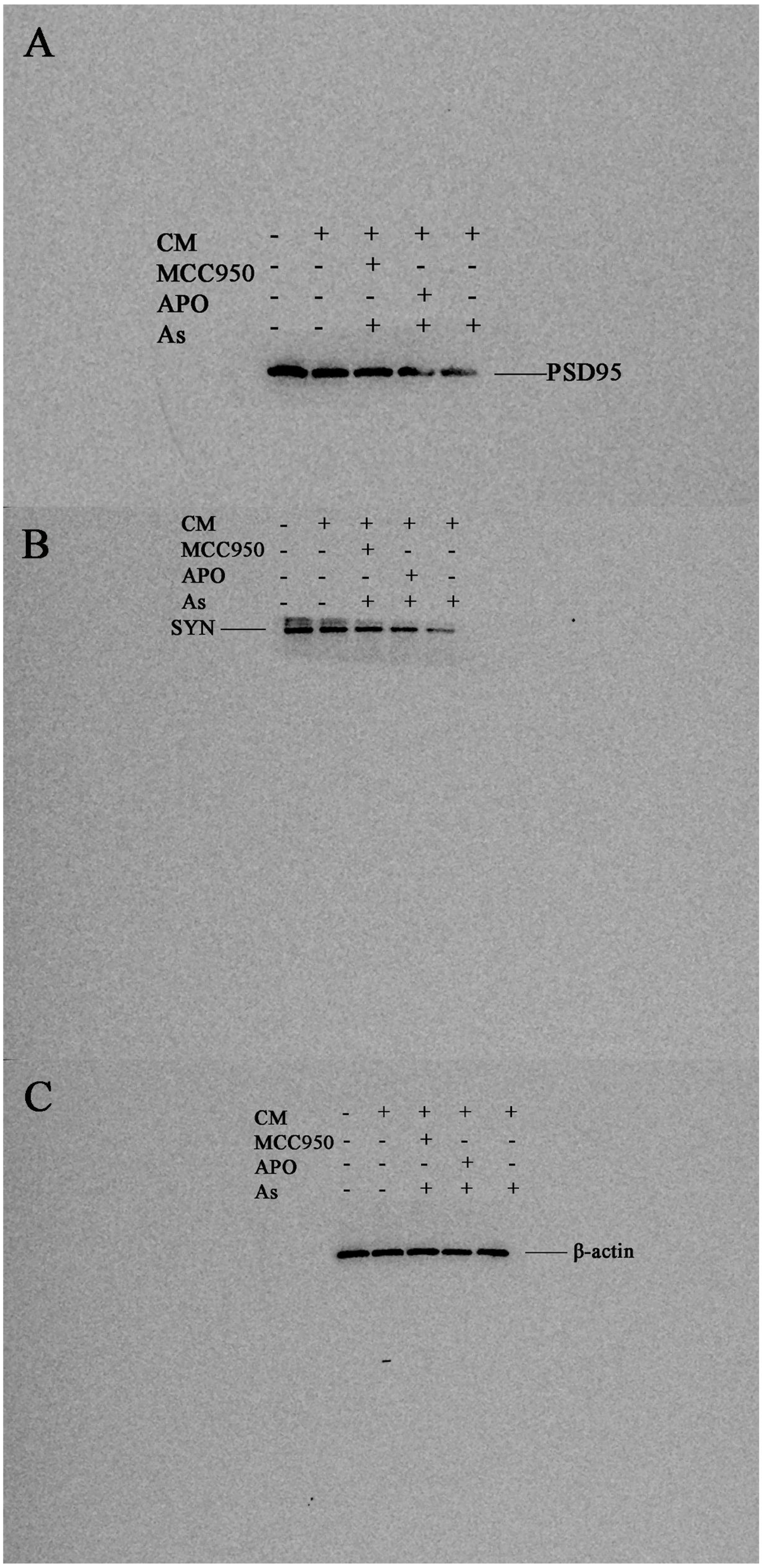

Figure S6. Full western blot images for Figure 6.
